# Sfrp1 as a Pivotal Paracrine Factor in the Trained Pericardial Stem Cells that Foster Reparative Activity

**DOI:** 10.1093/stcltm/szad075

**Published:** 2023-11-04

**Authors:** Hongtao Zhu, Xueqing Liu, Weili Ouyang, Yingcai Hao, Zheheng Ding, Kezhe Tan, Jianfeng Tang, Jianfeng Zhao, Xiaojun Ding, Zenghui Teng, Xiaoming Deng, Weidong Wu, Zhaoping Ding

**Affiliations:** Department of Cardiology, The People’s Hospital of Danyang affiliated to Nantong University, 212300 Danyang, People’s Republic of China; Department of Cardiology, The People’s Hospital of Danyang affiliated to Nantong University, 212300 Danyang, People’s Republic of China; Department of Cardiology, The People’s Hospital of Danyang affiliated to Nantong University, 212300 Danyang, People’s Republic of China; Department of Cardiology, The People’s Hospital of Danyang affiliated to Nantong University, 212300 Danyang, People’s Republic of China; Institute of Biochemistry and Molecular Biology II, Heinrich-Heine-University of Düsseldorf, Universitätsstr. 1, 40225 Düsseldorf, Germany; Department of General Surgery, Shanghai Children’s Hospital, School of Medicine, Shanghai Jiao Tong University, Shanghai, People’s Republic of China; Department of Cardiology, The People’s Hospital of Danyang affiliated to Nantong University, 212300 Danyang, People’s Republic of China; Department of Cardiology, The People’s Hospital of Danyang affiliated to Nantong University, 212300 Danyang, People’s Republic of China; Department of Cardiology, The People’s Hospital of Danyang affiliated to Nantong University, 212300 Danyang, People’s Republic of China; Institute of Neuro and Sensory Physiology, Heinrich-Heine University of Düsseldorf, 40225 Düsseldorf, Germany; Department of Anesthesiology, Changhai Hospital, Naval Medical University, Shanghai, People’s Republic of China; Department of Anesthesiology, The People’s Hospital of Danyang affiliated to Nantong University, 212300 Danyang, People’s Republic of China; Institute of Molecular Cardiology, Heinrich-Heine University of Düsseldorf, 40225 Düsseldorf, Germany

**Keywords:** anti-apoptosis, myogenic progenitors, Sfrp1, inflammatory niche, pericardial stromal cells

## Abstract

Tissue damage often induces local inflammation that in turn dictates a series of subsequential responses, such as stem cell activation and growth, to maintain tissue homeostasis. The aim of the study is to testify the possibility of using inflammation-trained stem cells as optimal donor cells to augment the efficacy of cell therapy. The pericardial stem/stromal cells derived from the animals after myocardial infarction (MI-pSC) showed an enhanced myogenic potential and augmented reparative activity after transplantation in the injured hearts, as compared to the Sham-pSC. Bulk RNA-Seq analysis revealed significant upregulation of a panel of myogenic and trophic genes in the MI-pSC and, notably, Sfrp1 as an important anti-apoptotic factor induced robustly in the MI-pSC. Injection of the MI-pSC yielded measurable numbers of surviving cardiomyocytes (Tunel and Casp-3 negative) within the infarct area, but the effects were significantly diminished by siRNA-based silence of Sfrp1 gene in the pSC. Primed Sham-pSC with pericardial fluid from MI rats mimicked the upregulation of Sfrp1 and enhanced myogenic potential and reparative activity of pSC. Taken together, our results illustrated the inflammation-trained pSC favor a reparative activity through upregulation of *Sfrp1* gene that confers anti-apoptotic activity in the injured cardiomyocytes. Therefore, the active form of stem cells may be used as a cardiac protective agent to boost therapeutical potential of stem cells.

## Graphical Abstract

**Graphical Abstract:**
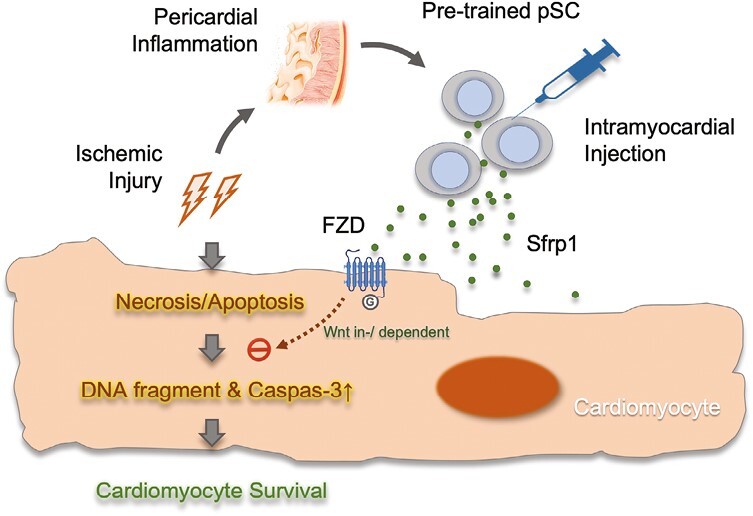

**Significance Statement**

While stem cell therapy holds immense promise to reconstruct the damaged hearts, the most clinical trials showed unmet success. The present experiments disclosed an active form tissue stem cells with enhanced myogenic potential and robust upregulation of trophic genes. Transplantation of inflammation-trained stem cells into the infarcted hearts boosted reparative activity via a Sfrp1-mediated anti-apoptotic action that salvages cardiomyocytes from ischemic damage. These results point out a therapeutical strategy of using naturally pretrained donor cells, or through genetic and pharmacological modification of Sfrp1 gene, to enhance the efficacy of cardiac stem cell therapy.

## Introduction

Cellular therapy continues to be pursued as an approach to reconstitute the damaged part of the heart after ischemic injury.^1,2^ However, the excitement in this burgeoning field has been somewhat dampened by some less than stellar clinical trial results and persistent variability of therapeutical effects. To overcome this challenge, a myriad of strategies that could putatively be exploited in stem cell modification have been conducted to improve outcomes in patients.^3^ These include pre-treating stem cells with hypoxia^4^ or cytokines,^5^ and using gene editing technologies to create stem cell derivatives with improved functionality, specificity and responsiveness compared with their natural counterparts.^6^ Notably, many of these take their inspiration from stem cell niche of the injury microenvironment in which quiescent stem cells are dramatically roused into an active form.^7^

The tissue niche is a specialized microenvironment in which a rapid and robust inflammatory response is triggered whenever tissue is injured, but it has also been co-opted to take on a huge variety of other roles during development, repair, and regeneration.^8^ In mammals, there is a need for an optimal level of innate and adaptive immune activation to maintain tissue homeostasis.^9^ Tissue stem cells are mostly resilient and remain quiescent in normal conditions but, upon inflammatory signaling in the setting of tissue injury, evolve into specialized and activated state to replenish exhausted or dead cells.^10^ Thus, activation of tissue stem cells mediates fundamentally natural tissue rejuvenation and performs regenerative acts prompted by injuries.^11^

In the view of cellular therapy, the donor cells applied in the most of preclinical and clinical studies were mainly the naturally occurring stem cells derived from normal tissue.^12^ However, the therapeutical potential of using naturally trained stem cells by factors in the inflammatory niche and, in particular, the action of mechanism of those cells that yield cardiac benefits have been poorly explored. In the present study, we characterized the inflammation-trained stem/stromal cells from the pericardial tissue after myocardial infarction (MI) and demonstrated a robust upregulation of a panel of myogenic and trophic genes. Furthermore, we identified the secreted frizzled-related protein-1 (Sfrp1) as pivotal paracrine factor that protected cardiomyocytes from ischemic injury and yielded cardiac structure repair and functional improvement after transplantation into the infarcted heart.

## Materials and Methods

### Animal Experiments

All the experiments were approved by the Institutional Animal Care and Use Committee at the Nantong University (NTU, S20200410-003) and conducted in accordance with standard operating guideline for animal care. Male Wistar rats (250-300 g, *n* = 32) and C57BL/6J (20-25 g, *n* = 28) used in the present study were bred at the animal center of NTU. All the animals were fed with a standard chow diet and received tap water ad libitum.

Experimental myocardial infarction (MI) was surgically induced by transiently occluding the left anterior descending artery (LAD) as previously described.^13^ After MI, intramyocardial injection (IM) was performed by using a U-100 insulin syringe of 30 G (Micro-Fine, BD). In each mouse, a total number of 100 000 cells suspended in 20 µL PBS were slowly injected within the infarcted ventricular-free wall at single spot. After injection, animals were allowed to recover until hemodynamics stabilized. Thereafter, chest was closed with one suture through the muscle and a second through the skin.

At various time points after operation indicated below, ie, 1 day for immune analysis; 5 days for pericardial stem/stromal cell isolation; and 28 days for in vivo differentiation and functional assessments, the animals were sacrificed with 100% CO_2_ in a 10-L glass chamber and the pericardial tissue together the heart was excised and transferred to ice-cold PBS. The heart together with the pericardial tissue was carefully positioned into tissue block (Tissue-Tek, Germany) and stored in −80 °C for histological analysis.

### Flow Cytometric Analysis of Pericardial Immune Cells

The analysis of immune cells was performed 1 day after operation both in the Sham (Sham-1D) and the MI (MI-1D) rats. In brief, the pericardial tissue was minced into small pieces with a scalpel and digested at 37 °C for 20 minutes (5 mL of 84 U/mL of Collagenase II, Biochrom, Beijing, China). The resulting cell suspension was further meshed through a cell strainer (40 μm, BD) to remove the undigested tissue pieces. Membrane epitope staining was performed as described previously.^14,15^ The antibodies for cytometric analysis were listed in Supplimentary Table S1. Cell composition was analyzed by a flow cytometer (FACS CantoII) in a specific gating strategy.

### Isolation of the Pericardial Stem/Stromal Cells

Isolation of the pericardial stem/stromal cells from the both rats and mice was performed 5 days after MI in the Sham- and MI-animals (Sham-5D and MI-5D, respectively) as previously reported.^14^ In brief, animals were sacrificed with 100 % CO_2_ and disinfected by transiently immersing the whole body into 75% ethanol solution (~1 minutes). Under a sterile condition, the pericardial tissue was carefully separated from the heart and surrounding tissues and minced into small pieces (~1 mm^3^). Digestive process was carried out within a 15-mL Flacon tube containing 5 mL of digestion solution (84 U/mL of collagenase II, Biochrom) with gently rotating (30 rpm) at 37 °C for 25 minutes. After centrifugation, cell pellet was re-suspended with MACS buffer (Miltenyi Biotec, Germany) and further purified by magnetic bead depletion to remove the cells positive for CD31 (endothelial cells) and CD45 (immune cells) with Mojosort nanobeads (BioLegend, San Diego, CA, USA) according to the manufacturer’s protocol.

The purified cells were seeded in a density of 2 × 10^3^/cm^2^ to T75 culture flask containing low sugar Dulbecco’s modified Eagle’s medium (DMEM, Sigma) supplemented with 30% FCS (PAN Biotech), penicillin (100 U/mL, Sigma), streptomycin (0.1 mg/mL, Sigma), and glutamine (1 mM, Sigma). Non-adherent cells were removed after initial plating in 24 h, and the adherent cells were then termed as pericardial stem/stromal cells (pSC) and cultivated at 37 °C with 5% CO_2_ for further experiments.

### Induction of Myogenic Differentiation

Myogenic induction was carried out when the isolated pSC reached sub-confluence (80%-90%, ~5-day cultivation). The inductive process was carried out simply by switching the culture medium into the low-serum condition that contained only 3% FCS + 5% horse serum, which efficiently reduced the proliferating rate of the cells and in parallel caused morphological changes characterized by elongation and cell fusion of the cultured cells. During the induction period, the medium was changed every 5 days for as long as 2 weeks when spontaneously beating activity appeared.

### Histology and Tunel Assay

Cryosections were made out of the tissue blocks in the thickness of 7 μm containing the heart and the pericardium. The sections were first fixed with 1% paraformaldehyde for 10 minutes and blocked with 5% normal goat serum (NGS) for 1 hour at room temperature. Primary antibodies were diluted according to the concentration of the individual antibodies as listed in Supplimentary Table S1. Immunostaining was carried out in a moist chamber at 4 °C overnight. After washing 3 times with PBS to remove the unbound antibodies, fluorochrome-conjugated secondary antibody (Cy3- or FITC-conjugated goat IgG, 1:100) was added and incubated at room temperature for 2 hours and nuclei were counterstained with 4ʹ,6-diamidin-2-phenylindol (DAPI, Sigma). Finally, the slides were carefully washed with PBS and mounted with Prolong Gold (ThermoFisher). Visualization of fluorescent images was digitalized by using a fluorescence microscopy (Olympus MX61) with software (CellSence) and analyzed with ImageJ (NIH, USA).

Immunocytochemistry was carried out as previously described.^16^ In brief, the cultured pSC at the 2nd passage were seeded on sterilized glass coverslips (24 × 24 mm) in a 6-wall plate and cultured for 3 days until 80% sub-confluence. The coverslips were then fixed with 1% paraformaldehyde and permeabilized with 0.01% Triton for 10 minutes at room temperature. Primary antibodies were added on the slips and incubated overnight at 4 °C. The slips were then washed for 3 times with 1% NGS-PBS buffer and incubated with either secondary antibody (Cy3- or FITC-conjugated goat IgG, 1:100) at room temperature for additional 60 minutes, counterstained with DAPI and sealed with mounting medium as described above. The percentage of the positive cells was the average of at least 5 independent fields of view at low magnification (10× object) and normalized to the total number of cells (DAPI positive).

Cardiac apoptosis was detected by a commercial kit from ABP Bioscience (TUNEL Andy Fluor 594 Apoptosis Detection Kit, A051) and performed according to manufacturer’s instruction. All images were digitalized and analyzed as described above.

In order to delineate the surviving cardiomyocytes within the infarct area where cell injection has been applied, we harvested the heart 24 hours after cell transplantation and infused triphenyl tetrazolium chloride (TTC) solution (1%, ThermoFisher) into the heart via an aortic cannula, in which TTC was enzymatically converted into insoluble formazan precipitates. The stained heart was then embedded in Tissue-Tek and sliced transversally at thickness of 50 µm with intervals of 200 µm from the apex to the site above injection of cells. As the formazan intensity correlates to the number of functional mitochondria seen mostly in viable cardiomyocytes, the crimson precipitates readily identify the surviving cardiomyocytes.

In the cell transplantation experiments, heart samples were obtained after functional assessment by MRI (see below) and embedded in Tissue-Tek . Successive cryosections (10 µm) were made from the apex to the site of LAD ligation with intervals of 200 µm. All slices were fixed with 4% paraformaldehyde for 10 minutes at room temperature and stained with 0.1% Sirius Red (SR, Direct Red 80, Sigma-Aldrich) for 5 minutes. Images were captured with a digital camera (UC30, Olympus) mounted on a microscope (MX 61, Olympus) and the thickness of the left anterior wall (LW thickness) was manually derived from the average of 6 independent radial distances between endocardial and epicardial plane around the site of injection.

### RNA-seq and Bioinformatic Analysis

The RNA sequencing (RNA-seq) analysis was commercially commissioned to Beyotime Biotech (Shanghai, China) and performed as previously described.^17^ In brief, total RNA was isolated from the primarily isolated and cultivated pSC (3-5 days after cultivation) using RNeasy mini kit (Qiagen, Germany) according to the manufacturer’s instructions. For the comparison, both pSC derived from the control (Sham) and the injured (MI) animals were included in the present analysis. The purified RNA samples (500 ng) were used for library construction and sequenced on the Illumina Hiseq PE150 to acquire a paired end-read (150 bp). The differentially expressed transcripts (DET) between 2 groups (Sham vs MI) were computed with *DESeq2* package (version 1.20.0) supplied by R software (version 3.4.4) via a likelihood ratio test implemented in *DESeq* function. Data were then subjected to functional enrichment analysis by STRING software (version 10.5).

### Expression Profiles of pSC

Based on the DET data from RNA-seq analysis, we selectively examined a panel of upregulated genes important to myogenic differentiation and tissue repair using quantitative real time PCR (qPCR) in the same RNA samples as for RNA-seq experiments as described previously.^15^ Results were presented as fold changes in relation to the expression levels of the Sham-pSC (referred as factor 1). Primers for target genes were commercially purchased (ThermoFisher) and listed in Supplimentary Table S2.

### RNA Interference of Sfrp1 Expression and ELISA Detection

To suppress Sfrp1 expression in the MI-pSC, siRNA-mediated gene silence was used. Briefly, siRNA oligonucleotides targeting Sfrp1 gene (5ʹ-GGCCAUCAUUGAACAUCUCtt-3ʹ and 5ʹ-GAGAUGUUCAAUGAUGGCCtt-3ʹ) and scramble siRNA (5ʹ-UUCUC CGAACGUGUCACGUtt-3ʹ and 5ʹ-ACGUGACACGU UCGGAGAAtt-3ʹ) were synthesized (GenePharma, China) and separately prepared at the concentration of 0.1 μM using in serum- and antibiotics-free DMEM. The mixture of siRNA and scramble siRNA together with transfection reagent (Lipofectamine 2000, Invitrogen, USA) was then added to the culture medium of MI-pSC and incubated at 37 °C overnight. Thereafter, the transfection medium was replaced by standard DMEM medium and ready for immunostaining and transplantation.

The concentration of soluble Sfrp1 in the culture medium was assessed by ELISA method according to manufacturer’s instruction (Invitrogen, USA)

### Assessment of Cardiac Function with MRI

Due to size-limitation of MRI coils for small animals, functional experiments were performed in mice for isograft cell transplantation. Magnetic resonance imaging (MRI) experiments were performed using a 400-MHz Bruker AVANCEIII 9.4-T wide-bore nuclear magnetic resonance spectrometer (Bruker, Germany) driven by ParaVision 5.1. Images were acquired with the Bruker Microimaging unit (Mini 0.5) equipped with a 30-mm birdcage resonator. Six to 8 contiguous ventricular short-axis slices (thickness 1 mm) of entire heart were acquired and cavity volume in each slice was obtained by multiplication of measured component area with slice thickness in either end-diastole or end-systole. Cardiac ejection fraction was calculated by dividing stroke volume (difference in end-diastolic and end-systolic volume) by the end-diastolic volume as reported previously.^15^

The infarct size was estimated by using late gadolinium enhancement (LGE) imaging 1 day post MI. Briefly, the mice were injected intraperitoneally contrast agent (Gd-DTPA, 0.1 mmol per kg BW) 10 minutes before measurements, and then a gradient echo sequence was performed to acquire the images of the LGE-positive areas of the heart sections. The infarct area was manually delineated with the Bruker Paravision region of interest (ROI) tool to validate all the animals showing a comparable infarct size.

### Collection of Pericardial Fluid and Myogenic Induction

Pericardial fluid (PF) was collected from the MI rats as reported previously.^16^ Briefly, in 5-day MI rats, the heart together the entire pericardial sac was first set free of the thoracic wall and carefully taken out with forceps. The pink fluid inside the pericardial cavity was collected into a prechilled 15 mL Falcon tube and centrifugated to remove blood cells. The supernatant (known as PF) was then aliquoted and stored at − 8 0°C for further analysis.

To test whether PF is able to induce the pSC into an activated state as in vivo condition, we administrated PF (30%) to the culture medium of sub-confluent Sham-pSC (+ PF), while the same amount of serum was used as the control (+ serum). After 5-day cultivation, cells underwent myogenic induction (low serum) to examine the differentiation potential by quantitative PCR or immunostaining as mentioned above. For transplantation experiments, pSC treated either with PF or serum as a control were injected into the infarcted heart and structural repair and functional improvement were analyzed 28 days post MI as mentioned above.

### Statistical Analysis

Data were presented as mean ± standard deviation. A Student’s *t* test with Welch’s correction was used to compare the expression of target genes and the fractions of positive cells and cardiac parameters of PF-treated mice. Comparison of LW thickness, apoptosis, vessel density, cross-sectional area, and MRI parameters cross groups were made with one-way analysis of variance (ANOVA) followed by Bonferroni *T* tests. Differences were considered significant at *P* < .05. The Prism software package (version 9.0) was used for the statistical analysis.

## Results

### Pericardial Inflammation Triggers Formation of Pericardial Progenitors

**Figure 1.**
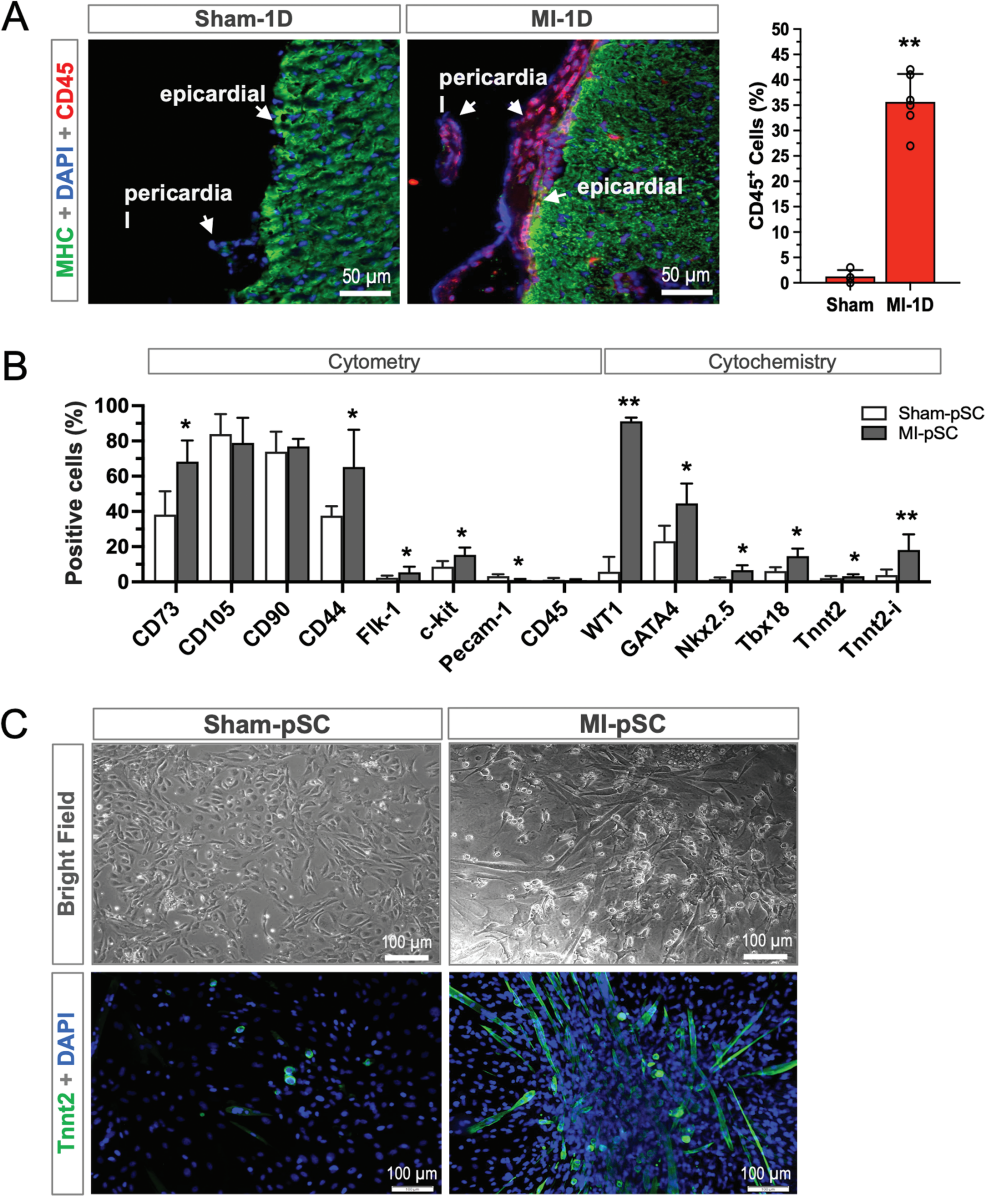
Pericardial inflammation after myocardial infarction led to the formation of myogenic stem/stromal cells. **A**. Immunostaining illustrated a robust immune cell infiltration (CD45^+^) in the epicardial and pericardial tissue within the first 24 hours after myocardial infarction (MI-1D), while the sham-operated hearts (Sham-1D) showed almost negative. Quantitative analysis by flow cytometry revealed the CD45^+^ cells proportionally surged up to 35.67% in the MI rats (*n* = 6), while only 1.25% (*n* = 4) in the Sham controls. **B**. Characterization of isolated pSC by either flow cytometry (surface makers) or cytochemistry (nuclear markers). The primary pSC showed mesenchymal features (CD73, CD90, CD105, and CD44) and significant increase in the proportion of several important transcriptional factors for cardiogenesis (WT1, GATA4, Nkx2.5, and Tbx18), as well as Tnnt2 after myogenic induction (Tnnt2-i) in the MI-pSC. Cellular contamination of endothelial (Pecam-1) and immune cells (CD45) was negligible after magnetic depletion. **C**. Microscopic analysis illustrated that, under low-serum cultivation, the MI-pSC elongated in cell size and formed significant number of cardiomyocyte-like phenotype (Tnnt2 positive) in contrast to the Sham-pSC. *P < .05 and **P < .01.

In the present experiments, we demonstrated that the MI-triggered inflammatory response was spread to the pericardial tissue, evidenced by robust infiltration of immune cells (CD45^+^ in Fig. 1A). The major composition of the infiltrated cells in the first 24 hours were pro-inflammatory monocytes (CD11b^+^/CCR2^+^, 64.14%, *n* = 6) and granulocytes (Gr^+^, 28.24%, *n* = 6), while the proportion of APC (CD11b^+^/MHC II^+^, 4.65%, *n* = 6) and lymphocytes (CD3^+^ and B220^+^, 1.43% and 1.01%, respectively, *n* = 6) was relatively modest. In contrast, almost no presence of CD45^+^ cells were detected within the myocardium, epicardium, and pericardium in the Sham-operated rats (Fig. 1A).

As inflammation may augment the reparative activity of tissue stem cells, we isolated the pericardial stromal fraction (pSC) from the MI rats (5 days post MI, MI-pSC) as well as from the sham-operated controls (Sham-pSC) to characterize and compare their properties by means of flow cytometric (surface makers) and cytochemical (nuclear markers) analysis. As expected, the pSC was relatively heterogenous population, but mostly showed positive staining for the makers of mesenchymal stem cells (CD73, CD90, CD105, and CD44) and small fraction for cardiac stem cell markers (Flk-1 and c-Kit), while contamination by endothelial (Pecam-1) and immune cells (CD45) was almost negligible after magnetic bead depletion (Fig. 1B). Interestingly, significant upregulation of the one of the well-known injury responsive elements, WT1, was found in the MI-pSC together with the slightly but significantly increased expression of several important transcriptional factors for cardiogenesis (GATA4, Nkx2.5, and Tbx18). Furthermore, Tnnt2 expression (cardiac specific) was implicitly upregulated in the isolated MI-pSC and became more evident after myogenic induction with low-serum cultivation (Tnnt2-i, Fig. 1B).

Myogenic induction entailed morphological alternation in the MI-pSC: they became elongated in size and always surrounded by multiple small, around cells, while the Sham-pSC showed typical cobblestone morphology as stromal cells (bright field in Fig. 1C), together with abundant Tnnt2 expressing cells found in the MI-pSC population (Fig. 1C, *P* < .01). The myogenic MI-pSC partially developed spontaneous beating activity in culture (Supplimentary Video S1). Therefore, the MI-pSC showed enhanced myogenic potential.

### Cardiac Reparative Activity of Pericardial Progenitors

**Figure 2.**
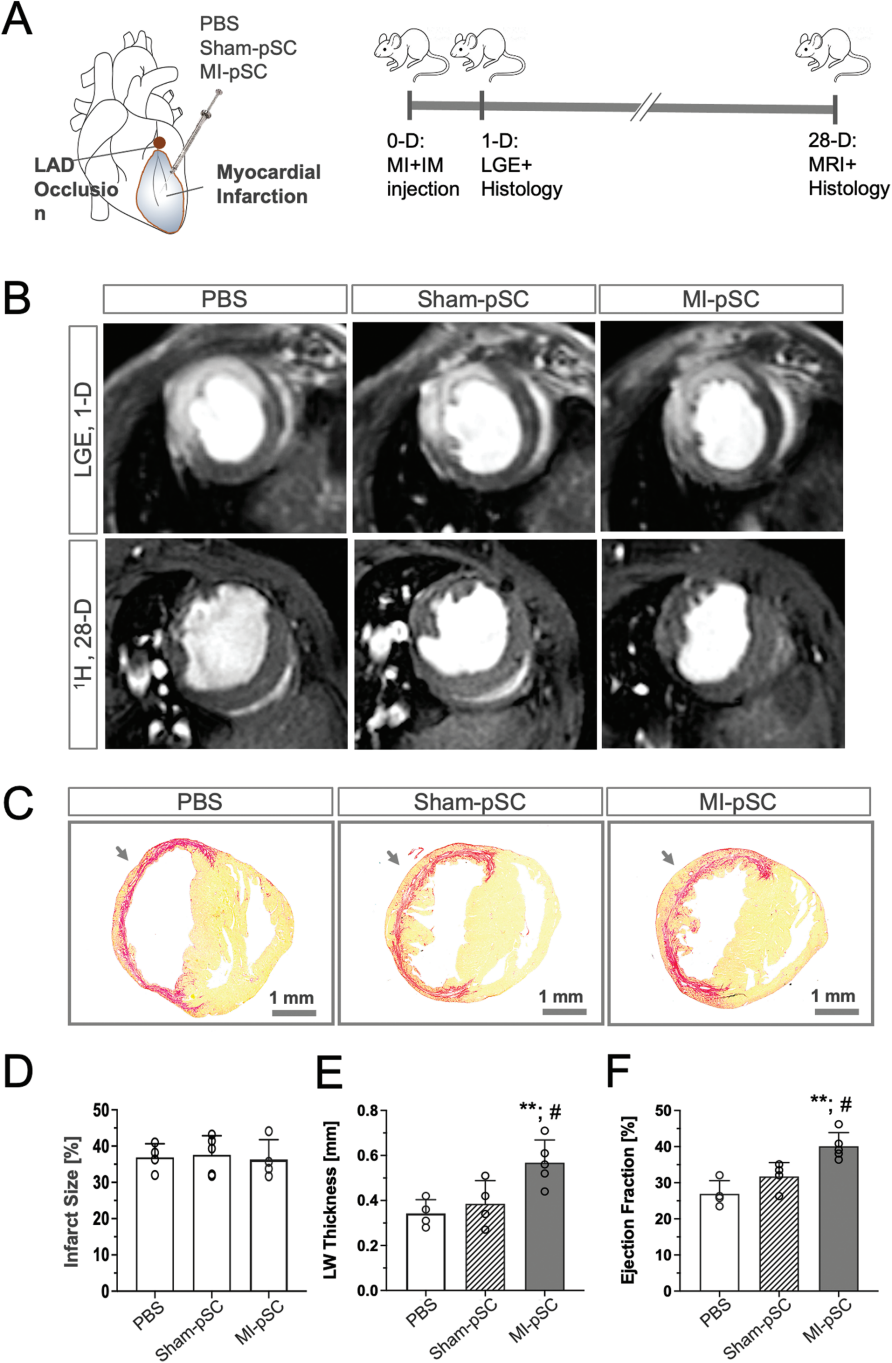
MI-pSC prevented cardiac deterioration in the infarcted hearts. **A**. Schematic drawing illustrated the experimental protocols of intramyocardial injection (IM) of pSC and PBS-control into the area of the infarcted wall. **B**. The infarct size was validated 1 day post MI (1D, *n* = 4-6) by late gadolinia enhancement (LGE), and cardiac function was assessed by MRI measurement 28 days post MI (28D, *n* = 4-6). **C**. Siriusred staining of the mid-ventricular sections of the 28D hearts showed fibrotic replacement of the infarcted area by collogens (red color) as well as in part cardiomyocytes (yellow). Arrow indicates the side of IM injection. **D**. Infarct size detected by LGE in 3 groups were comparable at the starting point (1D). **E** and **F**. Quantitative analysis of the left ventricular wall thickness (LW thickness) and ejection fraction reveled that injection of the MI-pSC (*n* = 5) yielded significant reconstitution of the infarcted wall and restoration of the contractile function of the infarcted hearts in comparison to the Sham-pSC treated animals (*n* = 4) as well as to the PBS controls (+PBS, *n* = 4). Arrows indicate the side of injection. #*P* < .01 in comparison to the control hearts. **P < .01 in comparison to the Sham-pSC-treated hearts.

To test the possibility to utilize the inflammation-trained pSC as therapeutical donor cells to mend the damaged heart, we transplanted MI-pSC into the infarcted heart and analyzed functional benefits (ejection fraction) and structural repair (left ventricular wall thickness, LW thickness) 28 days after injection. The same amount of 2 types of cells (Sham- and MI-pSC) were injected into the middle area of the infarcted ventricular wall, while PBS was used as controls (Fig. 2A). Experimental infarction in mice was comparable across 3 groups, shown by identical infarct size determined by LGE (Fig. 2B, D). Twenty-eight days after transplantation, while injection of the Sham-pSC showed a mild improvement of cardiac repair in comparison to PBS-controls, injection the MI-pSC yielded significantly more pronounced cardiac benefits in preventing dilative cardiomyopathies (wall thickness, Fig. 2B, 2E, *P* < .01), inducing angiogenesis (Supplimentary Fig. S1, *P* < .01), preventing compensatory hypertrophy (Supplimentary Fig. S1, *P* < .01) and in restoring contractile function (ejection fraction, Fig. 2B, 2F, *P* < .01), indicating the MI-pSC bears intrinsic activity that favors cardiac repair of the MI hearts.

### Globe Upregulation of Myogenic and Sfrp1 Genes in the MI-pSC

**Figure 3.**
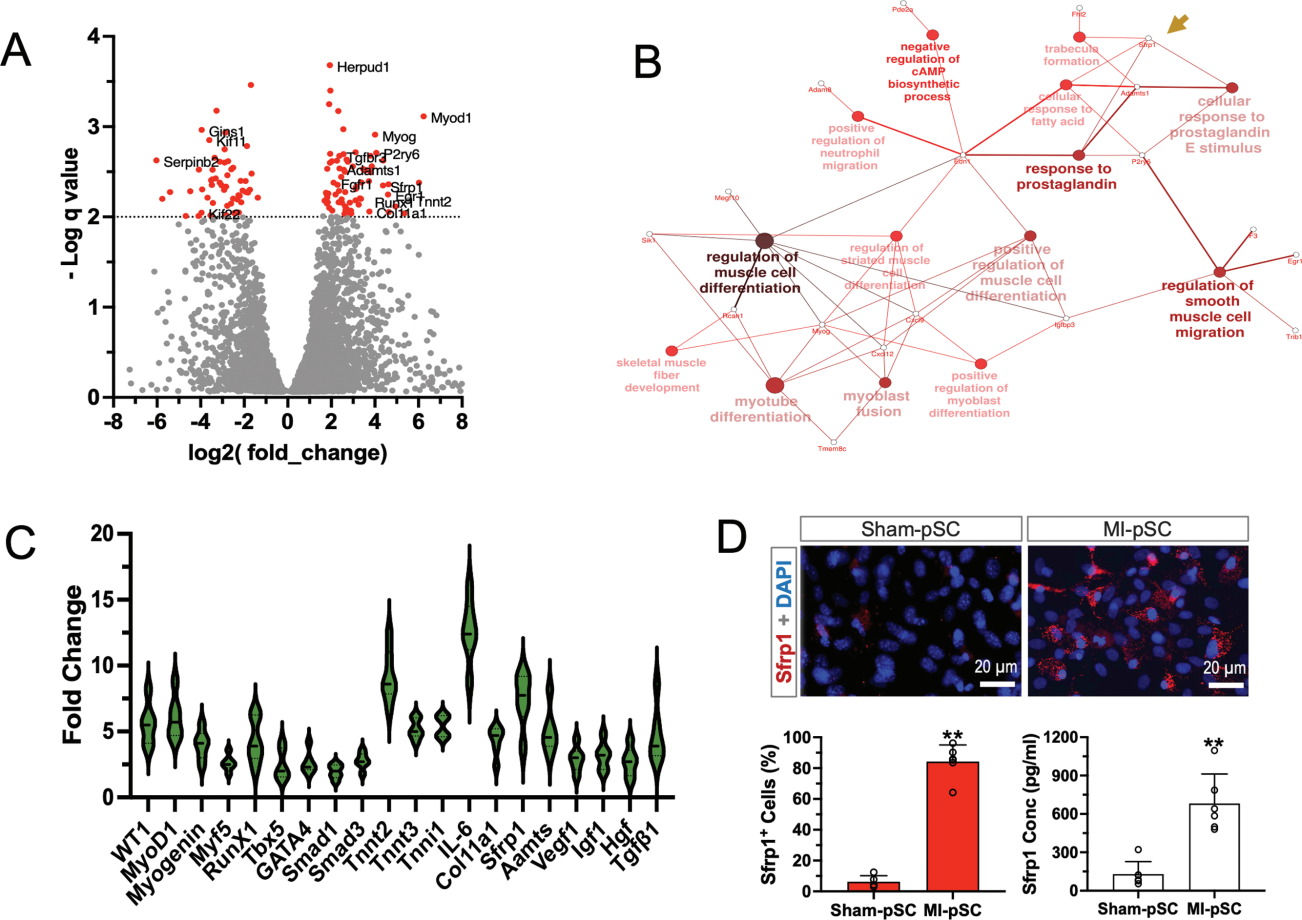
Enhanced myogenic differentiation and Sfrp1 upregulation in the MI-pSC. **A**. Volcano plot of the RNA-seq data showed the differentially expressed genes (DEG) between the Sham-pSC and MI-pSC populations. Note that a panel of myogenic genes were upregulated in the MI-pSC. **B**. Gene annotation and function analysis revealed the enhanced pathways related to myogenic differentiation, myoblast fusion and cellular response to prostaglandin E (Sfrp1, arrow). **C**. RT-PCR verified a panel of genes related to myogenic differentiation, tissue remodeling (growth factors and fibrosis) and anti-apoptotic process in the MI-pSC (*n* = 6). Data were presented as fold change in relation to the level of the Sham-pSC (referred as factor 1, *n* = 6). **D**. In the isolated and cultured pSC, Sfrp1-positive cells were found in the majority of the MI-pSC (*n* = 5), while significantly less in the Sham-pSC (*n* = 4). In line with the immunostaining data, Sfrp1 concentration detected by ELISA in the medium was found 5 times more abundant in the MI-pSC (*n* = 5) than the Sham-pSC. **P < .01.

We further analyzed the potential factors that mediate the reparative activity in the MI-pSC by using RNA deep sequencing. Total of 741 transcripts in the MI-pSC were found to be significantly upregulated and 394 downregulated as shown in the volcano plot (Fig. 3A). Gene annotation and function analysis revealed the enhanced pathways related to cardiac muscle tissue development (GO:0048738), regulation of muscle cell development (GO: 0054024) and cardiac cell differentiation (GO:0035051). Notably, cellular response to prostaglandin E was upregulated (Adamts1 and Sfrp1, arrow in Fig. 3B). We further verified a panel of genes related to myogenic differentiation, tissue remodeling (growth factors and fibrosis) and anti-apoptotic process in the MI-pSC, shown as fold change in relation to the level of the Sham-pSC (referred as factor 1, Fig. 3C). In addition to myogenic genes, MI-pSC also showed profound upregulation of genes for trophic factors, fibrotic and pro-inflammatory cytokines. In line with the mRNA data, abundant Sfrp1-positive cells were found in the cultured MI-pSC, while only modest in the Sham-pSC (Fig. 3D, *P* < .01). Furthermore, the Sfrp1 concentration detected by ELISA in the medium was found 5 times more abundant in the MI-pSC than the Sham-pSC (Fig. 3D, *P* < .01). Therefore, the stem/stromal cells within the inflammatory niche were likely shifted toward reparative phenotype by altering multiple genes related to tissue repair, in which Sfrp1 was likely a relevant factor linked to tissue homeostasis.

### Identification of Sfrp1 as an Anti-apoptotic Factor

**Figure 4.**
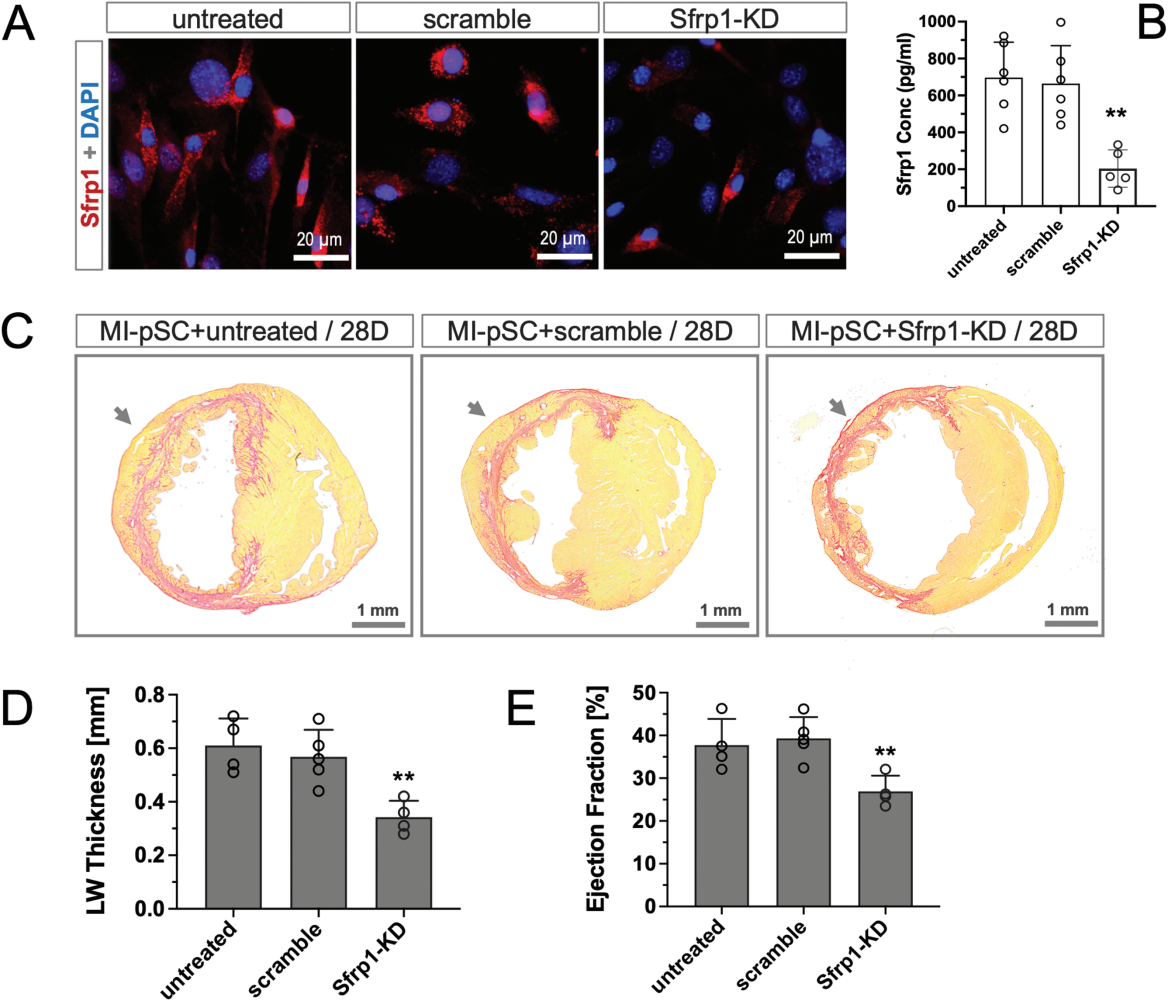
The essential role of Sfrp1 in cardiac repair induced by cell therapy. **A**. siRNA-based knockdown of Sfrp1 gene (Sfrp1-KD) reduced the proportion of Sfrp1 positive cells in comparison to the untreated and scrabble control of MI-pSC. **B**. Sfrp1 knockdown (*n* = 5) yielded 70% reduction of Sfrp1 concentration in the culture in comparison to the untreated and scrabble controls (*n* = 5-6). **C**. Siriusred staining of the 28-day hearts (28D) showed thickening of the ventricular wall in the site of injection, in which robust cardiomyocytes (yellow) abided within the fibrotic scar tissue (red). This effect was largely reduced when MI-pSC were lacking Sfrp1 expression (MI-pSC + Sfrp1-KD). **D** and **E**. Quantitative analysis showed the left ventricular wall thickness (LW thickness) and contractile function (ejection fraction) were significantly reduced in the Sfrp1-KD injected hearts (*n* = 4) as compared to the hearts received the untreated (*n* = 4) or scramble MI-pSC (*n* = 5). Arrow indicates the side of IM injection. **P < .01 in comparison to the Sham-pSC-treated hearts.

To examine the role of Sfrp1 mediating the reparative activity of MI-pSC, we used siRNA-based gene silence to knockdown Sfrp1 expression (Sfrp1-KD) in MI-pSC. Targeted suppression of Sfrp1 gene diminished the number of positive cells (Fig. 4A) and significantly reduced the secretion of Sfrp1 protein in culture medium as compared to the untreated and scramble controls (Fig. 4B, *P* < .01). Notably, myocardial injection of the Sfrp1-KD cells into the infarcted hearts yielded only a minor increase of the infarcted wall thickness, in direct contrast to the untreated or scramble cells that had intact Sfrp1 machinery and efficiently reconstituted the infarcted ventricular wall 28 days after transplantation (28D, Fig. 4C, 4D, *P* < .01). The compromised structural repair in the Sfrp1-KD-treated heart was associated with a minor functional improvement, as compared to the untreated or scramble controls (Fig. 4E, *P* < .01). These data therefore pointed to the MI-pSC-produced Sfrp1 as an important paracrine factor that yielded cardiac reparative effects in the MI hearts.

**Figure 5.**
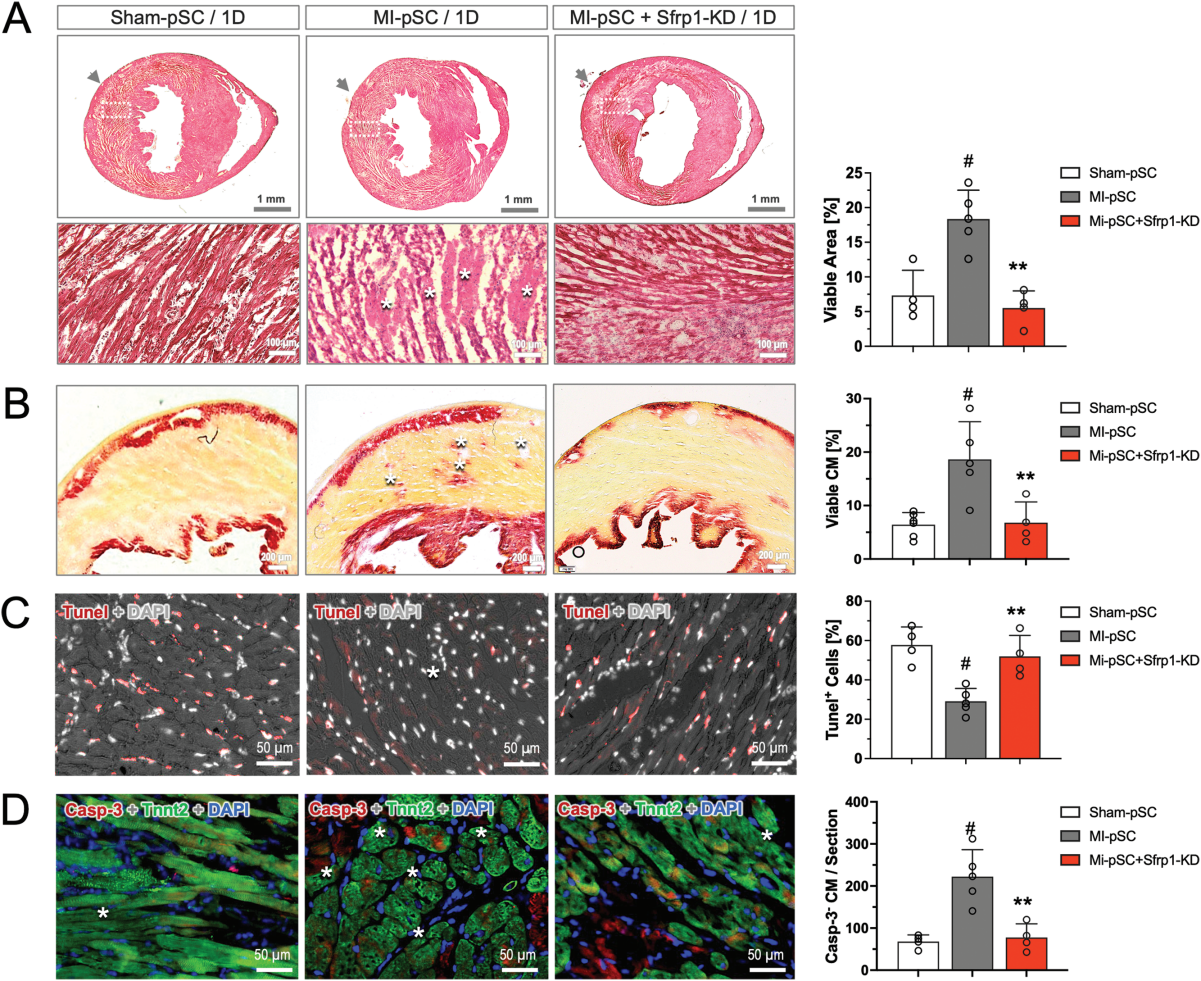
Sfrp1-mediated anti-apoptotic activity of MI-pSC. **A**. H&E staining of the 1D heart sections (1 day after transplantation of either Sham-pSC, MI-pSC, or MI-pSC + Sfrp1-KD) illustrated severe disruption of extracellular matrix and shrink of cardiomyocytes (CM) body in the infarct area. Note that, in contrast to the death CM, measurable surviving islands of CM (SI) within the infarct area evidenced by cellular integrity (asterisk). **B**. TTC staining confirmed the viability of cardiomyocytes, showing significantly abundant SI (asterisks) within the infract zone in the MI-pSC-injected hearts as compared to the Sham-pSC animals, but compromised in the Srfp1-KD animals. **C** and **D**. Most of cardiac cells in the infarct area underwent apoptotic cell death shown by DNA fragments (Tunel) and, in the cardiomyocytes, the presence of cleaved Caspase-3 (Casp-3). Quantitative analysis revealed that injection of MI-pSC (*n* = 5) significantly reduced apoptotic events and increased the numbers of Casp-3-negative CM (viable) as compared to the Sham-pSC treatment (*n* = 4). Suppression of Sfrp1 in the MI-pSC (*n* = 4) resulted in addition of Tunel^+^ cells and decline of viable CM (Casp-3 negative). Arrow indicates the side of injection. #*P* < .01 in comparison to the Sham-pSC-treated hearts and **P < .01 in comparison to the MI-pSC.

Given the potential cardioprotective effects of Sfrp1, we examined the anti-apoptotic mechanism underling the Sfrp1-mediated cardioprotection at the early stage. One day after transplantation (1D), H&E staining of the successive heart sections illustrated severe myocardial disruption and cell shrink, suggestive of necrotic/apoptotic cell death caused by ischemic injury (Fig. 5A). Interestingly, within the infarct area a measurable number of viable cardiomyocytes was detected, locating mainly in the area where pSC were applied. In contrast to the dead myocardium, the vital cardiomyocytes exhibited the integrity of cell membrane and existed as small clusters in the vicinity of injected cells (surviving islands, SI, asterisks in Fig. 5A). Quantitative analysis revealed that injection of the MI-pSC yielded most pronounced protective effects, in which 16% of myocardium within the infarct area were salvaged, while only 7% in the Sham-pSC injected hearts, while injection of the Sfrp1-supressed MI-pSC (MI-pSC + Srfp1-KD) caused ensuingly diminished protective effects, showing by the significantly reduced number of SI (right panel in Fig. 5A, *P* < .01). The cardiomyocytes viability was further confirmed TTC staining, showing significantly abundant SI within the infract zone in the MI-pSC-injected hearts as compared to the Sham-pSC animals (asterisks in Fig. 5B), but compromised in the Srfp1-KD animals (right panel in Fig. 5B, *P* < .01). The surviving cardiomyocytes were stained negative for Tunel (DNA fragment, Fig. 5C) and cleaved Caspase-3 that propagates an apoptotic signal through enzymatic activity on downstream targets, suggesting they have shielded and escaped from the ischemic insult (Fig. 5D). Quantitatively, increased proportion of the Tunel-positive cardiac cells (right panel in Fig. 5C, *P* < .01) and less Casp-3-negative cardiomyocytes (Casp-3^−^ CM, right panel in Fig. 5D), as compared to the naive MI-pSC. Therefore, cardiac injection of the MI-pSC was an effective cardioprotective intervention and caused by at least in part by Sfrp1-medated anti-apoptotic effects in the injured cardiomyocytes.

### Myogenic Induction by Pericardial Fluid

**Figure 6.**
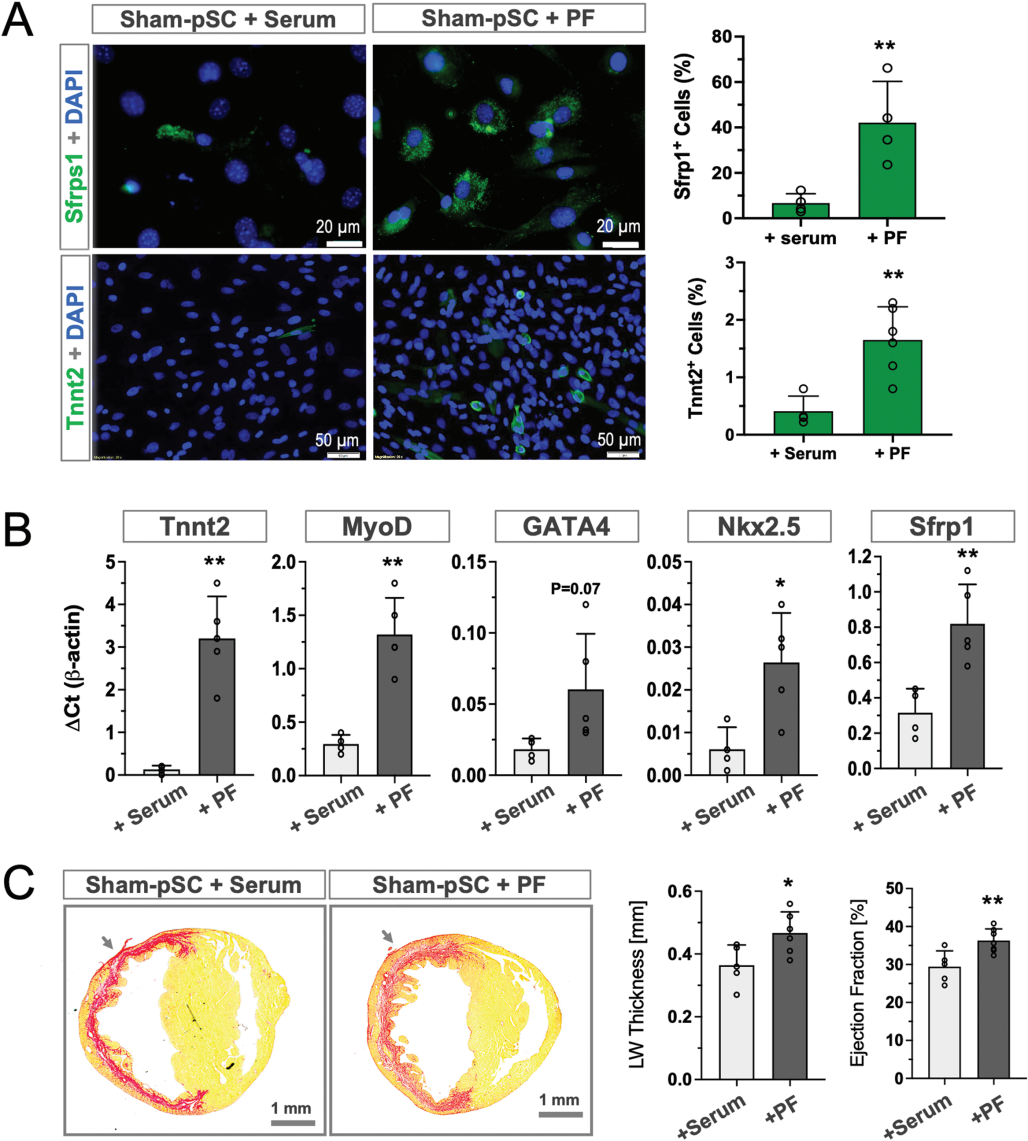
Pericardial fluid-primed pSC showed enhanced myogenic potential and reparative activity. **A**. The Sham-pSC primed with pericardial fluid (PF, 30%) in culture for 5 days significantly induced the formation of Sfrp1- and Tnnt2-positive cells (+PF, *n* = 5) in comparison to serum controls (+serum, *n* = 4). **B**. PF-treatment resulted in significant upregulation of Sfrp1, and a panel of genes related to cardiogenesis (*n* = 5). **C**. In vivo injection of PF-primed pSC (*n* = 7) into the infarcted heart yielded more pronounced efficacy in the reconstitution of the left ventricular wall (LW thickness) and in restoration of contractile function (ejection fraction) in relation to the Sham-pSC injected animals (*n* = 5). Arrow indicates the side of injection. *P < .05 and **P < .01.

We further tested whether the inflammatory niche could be mimicked by pericardial fluid (PF) to enhance myogenic potential and reparative activity in vitro condition. Taken the advantage of our sampling protocol, we were able to collect PF from the infarcted rats.^16^ As shown in the Fig. 6A, addition of PF (30%) in the culture medium resulted in a significant increase of MyoD and Tnnt2 expressing cells in comparison to the serum controls (*P* < .01). These results were further confirmed by RT-PCT analysis, showing a similar pattern of upregulation in MyoD- and Tnnt2-mRNA (Fig. 6B). Moreover, several cardiac genes as well as WT1 were found to be upregulated in the PF-treated cells (Fig. 6B). The induction was relatively less effective in view of in vivo condition, it nevertheless demonstrated the essential ability of the inflammatory signals to trigger the formation of myogenic progenitors in vitro. Notably, injection of the PF-treated pSC into the infarcted hearts yielded a significantly more pronounced ameliorative effects in the LW thickness and improvement of ejection fraction (Fig. 6C, *P* < .01), suggesting that PF was able to mimic the inflammatory niche in vitro condition and imposed an enhanced reparative activity to pSC.

## Discussion

In the present experiments, we demonstrated that inflammatory niche in the pericardial tissue triggered the formation of myogenic progenitors and a robust upregulation of Sfrp1 gene. After being transplanted into the infarcted heart, MI-pSC-produced Sfrp1 acted as a pivotal anti-apoptotic factor that salvaged cardiomyocytes from ischemic damage and created cardiac benefits of stem cell therapy. Therefore, our results point out the significance of cardioprotection that mediates the efficacy of cardiac stem cell therapy, which is enhanced by using inflammation-trained donor cells.

### Activated Stem Cells as Optimal Doner Cells

In the past 2 decades, naturally occurring stem cells, including hematopoietic and mesenchymal stem/stromal cells and, more recently, derivatives of pluripotent stem cells, have been widely used in cardiac cell therapy.^12^ Although several forms of cells have been beneficial in improving cardiac function,^18^ the outcome is mediocre, and it is still controversial as to whether they can truly regenerate lost myocardium. In the present study, we testified the possibility of utilizing the activated stem cells educated by inflammatory niche as donor cells to enhance the efficacy of cellular therapy.

Our previous experiments showed the pericardial tissue as a source of adipose stem cells that, upon MI, recapitulated one of the injury-responsive genes, WT1.^16^ However, action of mechanism by which the WT1-postive cells create cardiac benefits remains largely unknown. In the present experiments, we demonstrated a robust upregulation of a panel of myogenic and trophic genes and, in particular, Sfrp1 as anti-apoptotic factor upregulated by the inflamed microenvironment. Although it is not clear whether WT1 transcriptionally regulates Sfrp1 expression in the trained pSC, it is interesting to notice that both WT1 and Sfrp1 function as negative regulators of Wnt/β-catenin cascade that are involved in cardiac protective process^19^ (see below) as, in general, inflammation may lead to a series of adaptive changes in the stem cell population toward reparative phenotype in the maintaince of tissue homeostasis.^20^ Thus, activated stem cells are empowered at multiple levels with enhanced reparative activity and may serve as an optimal population for cell therapy. Particularly, because pericardial stem cell pool is developmentally derived from a pre-epicardial origin of mesenchymal cells^21^ and is abundant and easily accessible in the clinical setting, the activated pSC bears several merits for autologous cell therapy in MI patients.

### Activation of Resident Stem Cells by Inflammatory Niche

Inflammatory signaling is fundamentally crucial to bolster phenotypic fluidity of tissue stem cells in the course of tissue repair.^22,23^ In skeletal muscle, for instance, it is well known that satellite cells raise from a heterogeneous pool of dormant cells that, in response to tissue damage, acquire both stemness and multipotency in the healing process.^24,25^ As to the adult heart, the dormant epicardial cells could be reactivated into a healing state that fosters cardiac repair by cellular replacement^26^ or by a paracrine manner^27^ and also gained phagocytic activity.^28^ In the pericardium, the inflammatory response was likely linked to cardiac transudate that leaked from the injured heart into the pericardial sac to form pericardial fluid. We have previously collected cardiac transudate within the intact pericardial sac as biological samples for the assays of inflammatory cytokines.^16^ In the present study, we demonstrated that PF was sufficient to induce the dormant pSC into an active state with upregulated myogenic and Sfrp1 genes (Fig. 6C). Therefore, it is the first proof-of-concept evidence to demonstrate that dormant pSC were actuated by inflammatory factors into an active form in vitro, although the efficiency was relatively minor as compared to the in vivo conditions.

### Enhanced reparative activity was associated with Sfrp1 upregulation

While stem cell therapy holds immense promise, the obstacle still resides due to the unmet success in the most clinical trials.^12^ In the present study, we showed that the trained stem cells by the inflammatory niche may serve as optimal donor cells that favor an adaptive enhancement of reparative activity as to their natural counterparts (Fig. 2). The trained pSC showed global transcriptional alternation of many genes in the stem/stromal population, including myogenic, fibrotic, and trophic factors. Among the DEG genes shown in Fig. 3, we found Sfrp1 was particularly relevant to cardioprotective effects as reported previously.^29,30^ To this end, we analyzed the contribution of Sfrp1 in the reparative process of MI-pSC after cardiac transplantation. Sfrp1 was found significantly upregulated at mRNA and protein levels (Fig. 3C, 3D) and, remarkably, when Sfrp1 gene was knocked down, the MI-pSC-induced cardiac benefits were substantially hampered, suggesting the obligatory role of Sfrp1 that mediated the MI-pSC-induced cardiac structural repair and functional improvement.

The secreted frizzled-related protein (Sfrp) family consists of 5 secreted glycoproteins (Sfrp1-5) that shares a common cysteine-rich domain homologous to the putative Wnt-binding site of frizzled proteins. Sfrps are mainly induced by hypoxia and serum deprivation and function as an antagonist of Wnt/β-catenin cascade by forming an inhibitory complex with the Frizzled receptors (FZD).^31^ Previous studies have reported that Sfrp1 and 2, by the inhibition of canonical Wnt/β-catenin pathway in cardiomyocytes, protected cardiomyocytes from apoptosis during oxidative stress^32,33^ and augmented cardiac angiogenesis,^29^ suggesting Sfrps may act as protective factors in the course of tissue repair. Indeed, Sfrp2 has been identified as a specific paracrine factor generated by AKT overexpressing mesenchymal stem cells (MSC)^34^ and intracoronary infusion of MSC improved cardiac function by the anti-apoptotic action of paracrine factors (Galectin-3, Smad-5, Sfrp1, and Sfrp4).^35^

**Figure 7.**
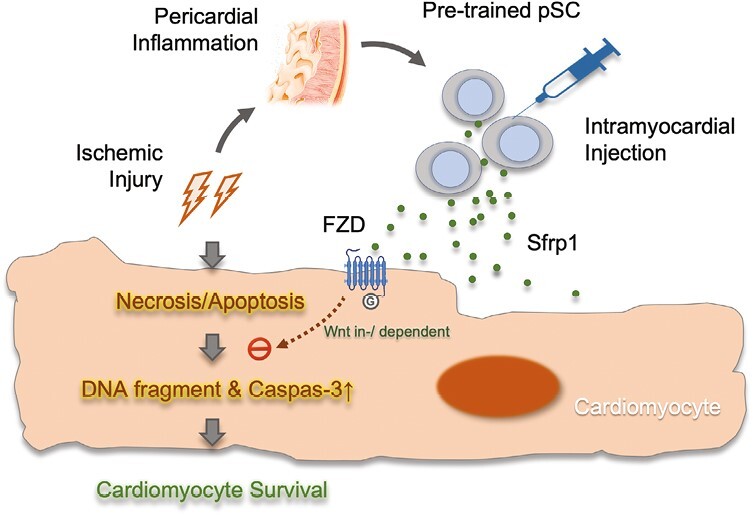
Schematic illustration of MI-pSC-mediated cardioprotection. The pericardial stem/stromal cells (pSC) are activated by inflammatory niche after myocardial injury and, after being transplanted into the injured myocardium, protect the cardiomyocytes from apoptotic cell death by enhanced release of secreted frizzled related protein 1 (Sfrp1). Sfrp1 binds to Wingless/Int1-Frizzled (FZD) receptor on cell membrane and, through Wnt-dependent and independent pathways, prevent injury-induced apoptosis and thus promote the survival of cardiomyocytes.

It is important to note that the injected cells almost disappeared with the first 3 days after transplantation and, therefore, analysis of the biological nature of paracrine factors should be restricted in the first 3 days after transplantation.^15^ Our experiment was the first time to demonstrate that, at the very early stage (within the first 24 hours), injection of the Sfrp1-enriched cell protected cardiomyocytes from ischemic injury in vivo conditions (Fig. 5). Therefore, it is possible, although highly hypothetical, that the Sfrp1-expressing cells produced locally extracellular Sfrp1 that bound to Wingless/Int1-Frizzled (FZD) receptor on cardiomyocytes and, through Wnt-dependent and/or independent mechanisms, prevented injury-induced apoptotic cascade and thus promoted the survival of cardiomyocytes. As illustrated in the present experiments (Fig. 5), the survived cardiomyocytes existed within the infarct area as small clusters (SI) in a close vicinity to the site of injection, highlighting the cellular communication via small molecules might be necessary to fulfill the beneficial effects (Fig. 7). Therefore, the present study provides direct evidence that stem cell-based therapy is indeed a cardiac protective intervention that, mediated by Sfrp1, salvages the cardiomyocytes from ischemic injury within the infarct zone.

## Conclusions

In summary, the present experiments debut the inflammation-trained stem cells in the pericardial tissue as attracting donor cells that, by up-regulating Sfrp1 gene, protect cardiomyocytes from ischemic injury. This finding points out stem cell-based therapy is indeed a cardiac protective intervention and, through genetic and pharmacological modification of Sfrp1 gene, the efficacy could be potentially augmented in the treatment of ischemic heart disease.

## Supplementary Material

szad075_suppl_Supplementary_Material

szad075_suppl_Supplementary_Video_S1

## Data Availability

Raw data and the necessary details can be provided by the corresponding authors under reasonable request.

## References

[1] Braunwald E. Cardiac cell therapy: a call for action. Eur Heart J. 2022;43(25):2352-2353. 10.1093/eurheartj/ehac188 35417529

[2] Marbán E. A phoenix rises from the ashes of cardiac cell therapy. Nat Rev Cardiol. 2021;18(11):743-744. 10.1038/s41569-021-00625-1 34556854

[3] Ahmad B , Skorska A, Wolfien M, et al. The effects of hypoxic preconditioned murine mesenchymal stem cells on post-infarct arrhythmias in the mouse model. Int J Mol Sci. 2022;23(16):8843. 10.3390/ijms23168843 36012110 PMC9408396

[4] Muscari C , Giordano E, Bonafè F, et al. Priming adult stem cells by hypoxic pretreatments for applications in regenerative medicine. J Biomed Sci. 2013;20(1):63. 10.1186/1423-0127-20-63 23985033 PMC3765890

[5] Abarbanell AM , Coffey AC, Fehrenbacher JW, et al. Proinflammatory cytokine effects on mesenchymal stem cell therapy for the ischemic heart. Ann Thorac Surg. 2009;88(3):1036-1043. 10.1016/j.athoracsur.2009.02.093 19699961

[6] Sano T , Ito T, Ishigami S, Bandaru S, Sano S. Intrinsic activation of cardiosphere-derived cells enhances myocardial repair. J Thorac Cardiovasc Surg. 2020;163(4):1479-1490.e5. 10.1016/j.jtcvs.2020.05.040 32682583

[7] Alagesan S , Brady J, Byrnes D, et al. Enhancement strategies for mesenchymal stem cells and related therapies. Stem Cell Res Ther. 2022;13(1):75. 10.1186/s13287-022-02747-w 35189962 PMC8860135

[8] Ginhoux F , Martin P. Insights into the role of immune cells in development and regeneration. Development. 2022;149(8):dev200829. 10.1242/dev.200829 35502783

[9] Epelman S , Liu PP, Mann DL. Role of innate and adaptive immune mechanisms in cardiac injury and repair. Nat Rev Immunol. 2015;15(2):117-129. 10.1038/nri3800 25614321 PMC4669103

[10] Mannino G , Russo C, Maugeri G, et al. Adult stem cell niches for tissue homeostasis. J Cell Physiol. 2022;237(1):239-257. 10.1002/jcp.30562 34435361 PMC9291197

[11] Fuchs E , Blau HM. Tissue stem cells: architects of their niches. Cell Stem Cell 2020;27(4):532-556. 10.1016/j.stem.2020.09.011 33007238 PMC7861346

[12] Gude NA , Sussman MA. Cardiac regenerative therapy: many paths to repair. Trends Cardiovasc Med. 2020;30(6):338-343. 10.1016/j.tcm.2019.08.009 31515053 PMC7050404

[13] Wang X , Zhang H, Nie L, et al. Myogenic differentiation and reparative activity of stromal cells derived from pericardial adipose in comparison to subcutaneous origin. Stem Cell Res Ther. 2014;5(4):92. 10.1186/scrt481 25084810 PMC4139604

[14] Tan K , Zhu H, Zhang J, et al. CD73 expression on mesenchymal stem cells dictates the reparative properties via its anti-inflammatory activity. Stem Cells Int. 2019;2019:1-12. 10.1155/2019/8717694 PMC6525959 31249602

[15] Ding Z , Tan K, Alter C, et al. Cardiac injection of USSC boosts remuscularization of the infarcted heart by shaping the T-cell response. J Mol Cell Cardiol. 2022;175:29-43. 36493853 10.1016/j.yjmcc.2022.11.006

[16] Tang J , Wang X, Tan K, et al. Injury-induced fetal reprogramming imparts multipotency and reparative properties to pericardial adipose stem cells. Stem Cell Res Ther. 2018;9(1):218. 10.1186/s13287-018-0959-1 30103817 PMC6090634

[17] Zhu H , Liu X, Ding Y, et al. IL-6 coaxes cellular dedifferentiation as a pro-regenerative intermediate that contributes to pericardial ADSC-induced cardiac repair. Stem Cell Res Ther. 2022;13(1):44. 10.1186/s13287-021-02675-1 35101092 PMC8802508

[18] Mastrolia I , Foppiani EM, Murgia A, et al. Challenges in clinical development of mesenchymal stromal/stem cells: concise review. Stem Cells Transl Med 2019;8(11):1135-1148. 10.1002/sctm.19-0044 31313507 PMC6811694

[19] von Gise A , Zhou B, Honor LB, et al. WT1 regulates epicardial epithelial to mesenchymal transition through β-catenin and retinoic acid signaling pathways. Dev Biol. 2011;356(2):421-431. 10.1016/j.ydbio.2011.05.668 21663736 PMC3147112

[20] Wang BJ , Alvarez R, Muliono A, et al. Adaptation within embryonic and neonatal heart environment reveals alternative fates for adult c‐kit ^+^ cardiac interstitial cells. Stem Cells Transl Med 2020;9(5):620-635. 10.1002/sctm.19-0277 31891237 PMC7180292

[21] Chong JJH , Chandrakanthan V, Xaymardan M, et al. Adult cardiac-resident MSC-like stem cells with a proepicardial origin. Cell Stem Cell 2011;9(6):527-540. 10.1016/j.stem.2011.10.002 22136928 PMC3652240

[22] Erler P , Monaghan JR. The link between injury-induced stress and regenerative phenomena: a cellular and genetic synopsis. Biochim Biophys Acta. 2015;1849(4):454-461. 10.1016/j.bbagrm.2014.07.021 25088176

[23] Lin B , Coleman JH, Peterson JN, et al. Injury induces endogenous reprogramming and dedifferentiation of neuronal progenitors to multipotency. Cell Stem Cell 2017;21(6):761-774.e5. 10.1016/j.stem.2017.09.008 29174332 PMC5722700

[24] Collins CA , Olsen I, Zammit PS, et al. Stem cell function, self-renewal, and behavioral heterogeneity of cells from the adult muscle satellite cell niche. Cell. 2005;122(2):289-301. 10.1016/j.cell.2005.05.010 16051152

[25] Howard EE , Pasiakos SM, Blesso CN, Fussell MA, Rodriguez NR. Divergent roles of inflammation in skeletal muscle recovery from injury. Front Physiol. 2020;11:87 doi: 10.3389/fphys.2020.00087. 32116792 PMC7031348

[26] Smart N , Bollini S, Dubé KN, et al. De novo cardiomyocytes from within the activated adult heart after injury. Nature. 2011;474(7353):640-644. 10.1038/nature10188 21654746 PMC3696525

[27] Zhou B , Honor LB, He H, et al. Adult mouse epicardium modulates myocardial injury by secreting paracrine factors. J Clin Invest. 2011;121(5):1894-1904. 10.1172/JCI45529 21505261 PMC3083761

[28] Ding Z , Temme S, Quast C, et al. Epicardium-derived cells formed after myocardial injury display phagocytic activity permitting in vivo labeling and tracking. Stem Cells Transl Med 2016;5(5):639-650. 10.5966/sctm.2015-0159 27057005 PMC4835243

[29] Dufourcq P , Descamps B, Tojais NF, et al. Secreted frizzled-related protein-1 enhances mesenchymal stem cell function in angiogenesis and contributes to neovessel maturation. Stem Cells. 2008;26(11):2991-3001. 10.1634/stemcells.2008-0372 18757297

[30] Han X , Amar S. Secreted frizzled-related protein 1 (SFRP1) protects fibroblasts from ceramide-induced apoptosis. J Biol Chem. 2004;279(4):2832-2840. 10.1074/jbc.M308102200 14581477

[31] Cruciat C-M , Niehrs C. Secreted and transmembrane Wnt inhibitors and activators. Cold Spring Harb Perspect Biol 2013;5(3):a015081-a015081. 10.1101/cshperspect.a015081 23085770 PMC3578365

[32] Hu Y , Guo Z, Lu J, et al. sFRP1 has a biphasic effect on doxorubicin-induced cardiotoxicity in a cellular location-dependent manner in NRCMs and rats. Arch Toxicol. 2019;93(2):533-546. 10.1007/s00204-018-2342-5 30377735

[33] Zhang Z , Deb A, Zhang Z, et al. Secreted frizzled related protein 2 protects cells from apoptosis by blocking the effect of canonical Wnt3a. J Mol Cell Cardiol. 2009;46(3):370-377. 10.1016/j.yjmcc.2008.11.016 19109969 PMC2710029

[34] Mirotsou M , Zhang Z, Deb A, et al. Secreted frizzled related protein 2 (Sfrp2) is the key Akt-mesenchymal stem cell-released paracrine factor mediating myocardial survival and repair. Proc Natl Acad Sci USA. 2007;104(5):1643-1648. 10.1073/pnas.0610024104 17251350 PMC1785280

[35] Nguyen B-K , Maltais S, Perrault LP, et al. Improved function and myocardial repair of infarcted heart by intracoronary injection of mesenchymal stem cell-derived growth factors. J Cardiovasc Transl Res 2010;3(5):547-558. 10.1007/s12265-010-9171-0 20559784

