# Supplementary material for: Sfrp1 as a Pivotal Paracrine Factor in the Trained Pericardial Stem Cells that Foster Reparative Activity
Source: Stem Cells Transl Med. 2023 Nov 4;13(2):137–50. doi: 10.1093/stcltm/szad075 (PMC10872698; doi:10.1093/stcltm/szad075)
Supplement: szad075_suppl_Supplementary_Material [file szad075_suppl_supplementary_material.pdf]

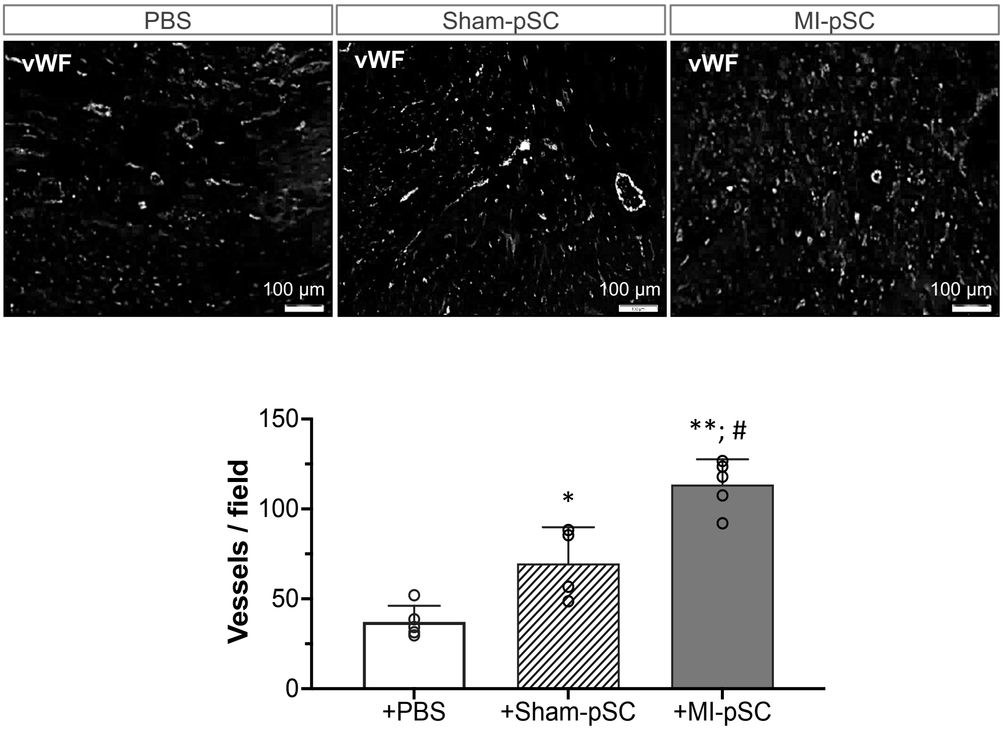

Supplemental Figure 1: Effects of pSC on angiogenesis. Heart sections (28-D after injection) were stained von Willebrand factor (vWF) as a highly selective endothelial marker that delineates the vascular density. Injection of pSC increased vessel density as compared to PBS-controls (n=5) and, remarkably, MI-pSC injection (n=5) yielded a more pronounced effect than Sham-pSC injection (n=4), suggesting angiogenic contribution to the MI-pSC-induced cardiac repair. * indicates $p<0.05$; ** indicates $p<0.01$ as compared to PBS controls. # indicates $p<0.01$ as compared to Sham-pSC.

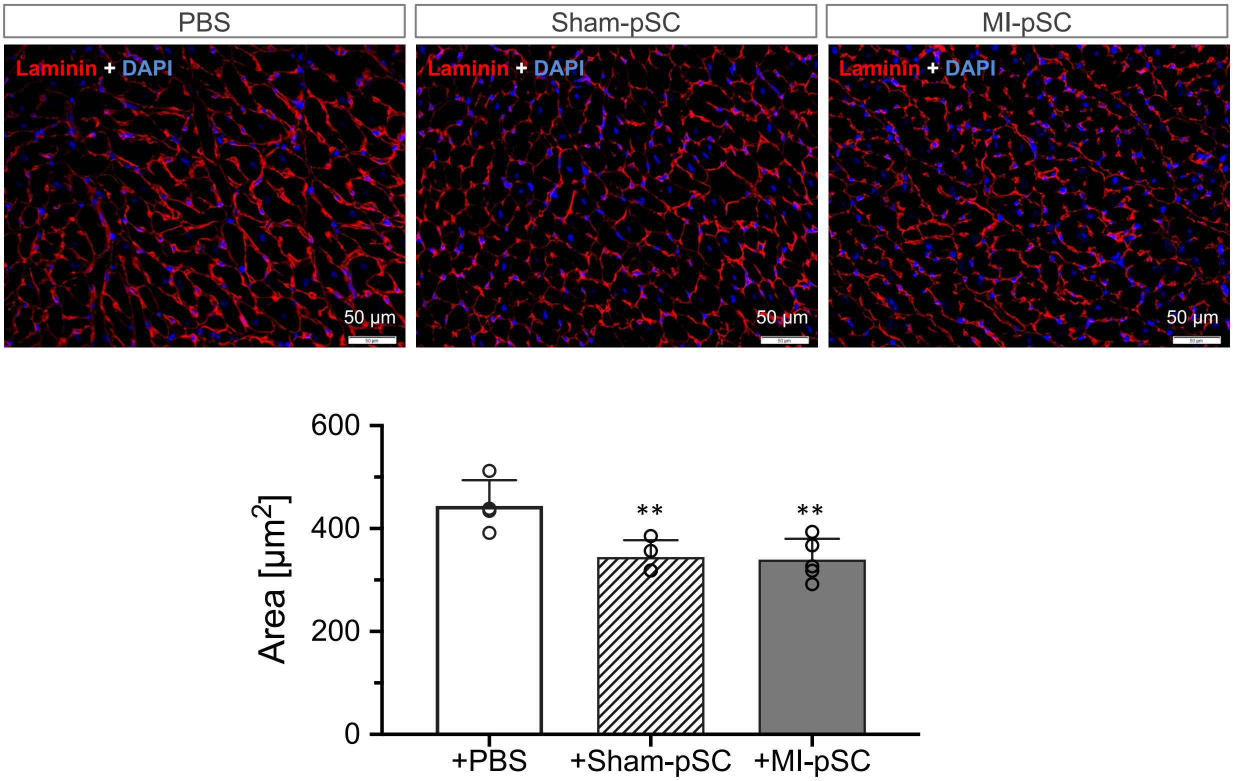

Supplemental Figure 2: Effects of pSC on myocytes hypertrophy. Heart sections (28-D after injection) were stained laminin as a cell membrane marker that delineates cross-sectional area of cardiomyocytes. Laminin clearly drew the outline of individual cells and only the area above 200 μm^2 were considered as myocytes by the add-in tool in ImageJ. Quantitative analysis was made from 5 individual sections of each heart and the results demonstrated that injection both Sham-pSC (n=4) and MI-pSC (n=5) showed smaller cross-sectional area of myocyte than the PBS controls (n=4), suggesting injection of pSC prevented compensatory hypertrophic responses in the infarcted hearts. ** indicates $p<0.01$ as compared to PBS controls.

Supplemental table 1: List of antibodies used in the present experiments

Antibodies	Host	Clonal	fluorochrome	Dilution	Source	Identifier	Applications
anti-MHC	rabbit	mono	none	1:400	Abcam	ab37484	Histology
anti-Tnnt2	rabbit	poly	none	1:400	Thermo Fisher	MS-295-P1	Histology
anti-Sfrp1	rabbit	poly	none	1:200	Merck	HPA064870	Histology
anti-Casp-3	rabbit	poly	none	1:200	Merck	AB3625	Histology
anti-CD45 (IBL-5/25)	rabbit	mono	none	1:400	ThermoFisher	ab203383	Histology
anti-WT1 (C-19)	rabbit	mono	none	1:200	Santa Cruz	SC-192	Histology
anti-GATA4 (G4)	rabbit	mono	none	1:400	Santa Cruz	SC-25310	Histology
anti-Tbx18 (CD21)	rabbit	mono	none	1:200	Santa Cruz	SC-130428	Histology
anti-Nkx 2.5	rabbit	mono	none	1:200	Santa Cruz	SC-376565	Histology
anti-mouse IgG	goat	poly	FITC	1:200	Abcam	ab97050	Histology
anti-rabbit IgG	goat	poly	TRITC	1:400	Abcam	ab6718	Histology
anti-laminin	rabbit	poly	none	1:200	Abcam	ab11575	Histology
anti-vWF	rabbit	poly	none	1:200	Dako	A00082	Histology
anti-CD32	mouse	mono	none	1:800	BD	550270	Flow Cytometry
anti-CD45	mouse	mono	PE	1:800	BD	554878	Flow Cytometry
anti-CD3	mouse	mono	APC	1:500	BD	557030	Flow Cytometry
anti-CCR2	rabbit	poly	none	1:200	Invitrogen	PA523037	Flow Cytometry
anti-CD45R (B220)	mouse	mono	FITC	1:200	eBioscience	11-0460-82	Flow Cytometry
anti-CD11b/c	mouse	mono	eFluor660	1:500	eBioscience	50-0110-80	Flow Cytometry
anti-MHC (M5/114 .15.2)	mouse	mono	FITC	1:400	LS Bio	LS-C107038	Flow Cytometry
anti-CD73	mouse	mono	PE	1:200	BD	554878	Flow Cytometry
anti-CD105	mouse	mono	Alexa 647	1:500	Bioss	50-0110-80	Flow Cytometry
anti-CD90	mouse	mono	FITC	1:200	eBioscience	12-0570-80	Flow Cytometry
anti-CD44 (OX-50)	mouse	mono	FITC	1:200	Invitrogen	MA516910	Flow Cytometry
anti-c-Kit (CD117)	mouse	mono	PE-Cyanine7	1:200	eBioscience	11-0460-82	Flow Cytometry
anti-Flk-1 (CD309)	rabbit	mono	none	1:400	Santa Cruz	SC504	Flow Cytometry
anti-Pecam-1	rabbit	poly	none	1:100	Millipore	MAB1393Z	Flow Cytometry

Supplemental table 2: List of Taqman primers used in the present experiments

	Targets (full name)	Abbreviation	Indentifier
1	Wilm's tumor factor 1	WT1	Rn00580566_m1
2	myoblast determination protein 1	MyoD	Rn00580555_m1
3	myogenin	Myogenin	Rn00567418_m1
4	myogenic factor 5	Myf5	Rn01502778_m1
5	SMAD family member 1	Smad1	Rn00565555_m1
6	SMAD family member 3	Smad3	Rn00565331_m1
7	troponin type 3	Tnnt3	Rn01645281_m1
8	troponin type 2	Tnnt2	Rn00562059_m1
9	GATA Binding Protein 4	GATA4	Rn01530459_m1
10	collagen Type XI Alpha 1 Chain	Col11a1	Rn01523309_m1
11	Runt related transcription factor 1	RunX1	Rn01645281_m1
12	T-Box transcription factor 5	TBX5	Rn01481891_m1
13	secreted frizzled-related protein 1	Sfrp1	Rn01478472_m1
14	ADAM metallopeptidase with thrombospondin type 1	Aamts1	Rn01445711_m1
15	vascular endothelial growth factor A	Vegf1	Rn01511602_m1
16	insulin-like growth factor 1	Igf1	Rn00710306_m1
17	hepatocyte growth factor	Hgf	Rn00566673_m1
18	ransforming growth factor, beta 1	Tgfbeta1	Rn00572010_m1
19	glyceraldehyde-3-phosphate dehydrogenase	GAPDH	Rn01775763_g1
20	β-actin	ACTB	Rn00667869_m1
